# Identification of candidate genes on the basis of SNP by time-lagged heat stress interactions for milk production traits in German Holstein cattle

**DOI:** 10.1371/journal.pone.0258216

**Published:** 2021-10-14

**Authors:** Kathrin Halli, Seyi Fridaius Vanvanhossou, Mehdi Bohlouli, Sven König, Tong Yin

**Affiliations:** Institute of Animal Breeding and Genetics, Justus-Liebig-University Gießen, Gießen, Germany; Humboldt-Universitat zu Berlin, GERMANY

## Abstract

The aim of this study was to estimate genotype by time-lagged heat stress (HS) variance components as well as main and interaction SNP-marker effects for maternal HS during the last eight weeks of cow pregnancy, considering milk production traits recorded in the offspring generation. The HS indicator was the temperature humidity index (THI) for each week. A dummy variable with the code = 1 for the respective week for THI ≥ 60 indicated HS, otherwise, for no HS, the code = 0 was assigned. The dataset included test-day and lactation production traits from 14,188 genotyped first parity Holstein cows. After genotype quality control, 41,139 SNP markers remained for the genomic analyses. Genomic animal models without (model VC_nHS) and with in-utero HS effects (model VC_wHS) were applied to estimate variance components. Accordingly, for genome-wide associations, models GWA_nHS and GWA_wHS, respectively, were applied to estimate main and interaction SNP effects. Common genomic and residual variances for the same traits were very similar from models VC_nHS and VC_wHS. Genotype by HS interaction variances varied, depending on the week with in-utero HS. Among all traits, lactation milk yield with HS from week 5 displayed the largest proportion for interaction variances (0.07). For main effects from model GWA_wHS, 380 SNPs were suggestively associated with all production traits. For the SNP interaction effects from model GWA_wHS, we identified 31 suggestive SNPs, which were located in close distance to 62 potential candidate genes. The inferred candidate genes have various biological functions, including mechanisms of immune response, growth processes and disease resistance. Two biological processes excessively represented in the overrepresentation tests addressed lymphocyte and monocyte chemotaxis, ultimately affecting immune response. The modelling approach considering time-lagged genotype by HS interactions for production traits inferred physiological mechanisms being associated with health and immunity, enabling improvements in selection of robust animals.

## Introduction

The tripled number of days with extreme temperatures in European countries from 1950 to 2018 [1] indicates the increasing importance of studying the effect of climate change on primary and functional traits in dairy cattle. Comparing to other domesticated livestock species, high productive Holstein Friesian cattle responded very sensitive to heat stress (HS), especially during the challenging early lactation period with temperatures beyond the thermoneutral zone lower than 5°C or higher than 25°C [2]. Selection on improved milk production simultaneously increased feed intake and body weight, implying a raised metabolic heat increment and a decline of the thermoneutral temperature range [3]. Furthermore, in addition to temperature, dairy cow trait alterations were influenced by humidity, suggesting consideration of a temperature-humidity index (THI) in genetic HS studies [4].

Response to direct HS, i.e., HS close to the trait recording date, was observed when THI exceeds a certain threshold. For example, in Holstein cows, protein yield decreased at THI 68 [5]. Genetically, random regression models were applied to infer genetic (co)variance components along a continuous THI scale [6]. Such modelling approach was also used to detect possible genotype x environment interactions (GxE), and THI dependent re-rankings of sires have been observed [7]. Genomically, genetic markers significantly associated with HS response and underlying candidate genes were detected via genome-wide association studies (GWAS). Four single nucleotide polymorphisms (SNPs) contributing to a milk yield decline under HS were identified by Hayes et al. [8] in Australian Holstein Friesian cattle. The four SNPs were located on *Bos taurus* autosomes (BTA) 8, 10, 25, and 29. Among them, the SNP *BFGL-NGS-30169* on BTA29 was also identified in Jersey cattle, and the strongest annotated candidate gene for the variation of milk yield under HS was the fibroblast growth factor 4 [8]. Selection of cattle displaying the ability to regulate body temperature under HS will contribute to improved heat resistance genetically, implying only marginal detrimental effects on primary and functional traits. Thus, the genetic architecture of traits reflecting the thermoregulation ability under HS, such as rectal temperature and respiration rate, was investigated in Holstein and Gir x Holstein crossbred cattle [9, 10]. Rectal temperature was used an indicator for HS [11], and respiration rate reflects the ability to maintain body temperature through evaporative cooling [10].

Most of the genetic studies focused on immediate responses to HS, i.e., considering THI at the measuring day, or the average THI shortly before the measuring day. Phenotypically, HS during late gestation in dams, termed as maternal or in-utero HS, significantly impaired growth, metabolism, immune functions and survival in offspring [12–14], since this period plays a crucial role in fetal growth [15]. Monteiro et al. [13] studied long-term effects of in-utero HS on female fertility and performance traits recorded in offspring and identified most detrimental impact of HS from the last 6 weeks of gestation. Quantitative-genetically, Halli et al. [16] identified alterations of genetic co(variance) components for weight and growth traits of dual-purpose cattle due to HS impact on their dams during the late pregnancy period.

In this regard, the current study focusses on enhanced genomic modelling approaches to inferring time-lagged HS impact, and considers a large dataset of genotyped Holstein Friesian cows kept in large-scale contract herds. Specifically, we firstly aim on the detection of significant SNP for the direct (main) and the interaction effect in the context of time-lagged HS on milk production traits at the first official test-day after calving and on first lactation production records. The main effect represents the SNP effect for the respective trait expressed consistently, independent from time-lagged HS. The interaction component is the difference of the SNP effects between cows undergoing in-utero HS or not. Afterwards, on the basis of the identified SNP associations, we annotated potential candidate genes. Against this background, i.e., in the context of pronounced time-lagged HS, we postulate the identification of candidate genes affecting overall robustness, health and adaptation.

## Materials and methods

### Cow traits

The official records at the first test-day for the milk production traits included milk yield (TMY), fat percentage (TFP), fat yield (TFY), protein percentage (TPP), protein yield (TPY) and somatic cell score (TSCS). First lactation records were available for milk yield (LMY), fat percentage (LFP), fat yield (LFY), protein percentage (LPP) and protein yield (LPY). The first test-day records of the genotyped cows covered the period between 5 and 35 days after calving. Lactation lengths for LMY, LFP, LFY, LPP and LPY comprised 275 to 305 days in milk. Age at first calving ranged from 20 to 40 months, and calving years from 2010 to 2015. A phenotypic dataset including 14,188 first parity Holstein cows was available for subsequent GWAS. The cows are kept in 53 large-scale herds, located in the German federal states of Mecklenburg-West Pomerania and Berlin-Brandenburg. The average number of cows per herd ranged from 55 to 775. For the test-day records, cows with extreme TMY (≤ 2 kg per day or ≥ 60 kg per day) and TSCS larger than 9,999,000 cells/ml, were excluded. Descriptive statistics for the test-day and the lactation production traits are listed in Table 1.

**Table 1:** Descriptive statistics of first test-day and lactation production traits.

Trait^a^	#observations	Mean	Minimum	Maximum	Standard deviation
**TMY**	12,333	28.78	2.00	58.40	6.31
**TFP**	12,333	4.14	1.62	10.17	0.78
**TFY**	12,333	1.18	0.07	3.62	0.28
**TPP**	12,333	3.24	2.14	5.67	0.33
**TPY**	12,333	0.92	0.09	1.90	0.18
**TSCS**	12,307	2.80	-1.06	9.64	1.67
**LMY**	12,804	9104.29	1994.00	16156.00	1476.15
**LFP**	12,804	3.82	2.21	5.99	0.45
**LFY**	12,804	345.92	90.00	571.00	51.33
**LPP**	12,804	3.35	2.64	4.16	0.20
**LPY**	12,804	303.37	68.00	528.00	44.44

^a^: TMY = first test-day milk yield; TFP = first test-day fat percentage; TFY = first test-day fat yield; TPP = first test-day protein percentage; TPY = first test-day protein yield; TSCS = first test-day somatic cell score; LMY = first lactation milk yield; LFP = first lactation fat percentage; LFY = first lactation fat yield; LPP = first lactation protein percentage; LPY = first lactation protein yield.

### Genotype data

The cows were genotyped using the *Illumina BovineSNP50 v2 BeadChip* (3,775 animals), or the *Illumina Bovine Eurogenomics 10K low-density chip* (10,413 animals). The 10K SNP genotypes were imputed to the 50K SNP panel by project partner vit (Verden, Germany), as implemented in the process for national routine genetic evaluations [17]. SNP quality controls were performed using the preGSf90 program from the BLUPf90 package [18, 19]. Filtering criteria for markers were as follows: consideration only of SNPs located on *Bos taurus* autosomes, minor allele frequency larger than 0.01, minimum animal and SNP call rate of 0.95 and no significant deviation from Hardy-Weinberg equilibrium (the difference between observed and expected heterozygous frequencies was smaller than 0.15). Furthermore, we deleted cows pairs with genomic relationships [20] larger than 0.95. Finally, 41,139 SNPs from the 14,188 genotyped cows were used for the ongoing genomic analyses. The test-day data were available from 12,333 genotyped cows, and 12,804 genotyped cows had first lactation records. In total, 10,949 genotyped cows had both test-day and lactation records. The SNP positions were coordinated according to the reference assembly ARS-UCD1.2.

### Meteorological data

Pairwise distances (in km) were calculated between weather stations and cow herds. The calculation based on coordinates for the respective longitude and latitude of each herd and weather station, and was performed using the GEOSPHERE package in R [21]. According to the minimal distances, we allocated 32 weather stations to 53 herds. The maximum distance between a herd and a weather station was 27.88 km, the minimum distance was 0.74 km and the average distance was 14.79 km. Hourly THI were calculated considering hourly temperature (T) and relative humidity (RH) as follows [22]:

THI=[1.8×T+32]−[0.55−0.0055×RH]×[1.8×T−26]

The daily THI was computed by averaging hourly THI over 24 hours. Afterwards, we calculated the average daily THI within the following weeks during late gestation of the respective dam, or, in other words, before the birth date of the genotyped cows: 0–7 days (WK1), 8–14 days (WK2), 15–21 days (WK3), 22–28 days (WK4), 29–35 days (WK5), 36–42 days (WK6), 43–49 days (WK7) and 50–56 days (WK8). The range of the weekly THI across the eight weeks was from 10.43 to 73.10. Descriptive statistics for the weekly THI are displayed in Table 2.

**Table 2:** Descriptive statistics of the weekly temperature humidity index before birth.

Week^a^	Mean	Minimum	Maximum	Standard deviation
**1**	47.88	11.47	72.44	12.10
**2**	47.83	11.80	73.10	12.22
**3**	47.84	10.43	72.52	12.16
**4**	47.91	14.02	72.62	12.10
**5**	48.01	10.43	72.64	11.95
**6**	48.08	11.76	72.62	11.99
**7**	48.10	11.76	72.33	12.03
**8**	48.17	14.40	73.10	11.99

^a^: week before birth.

### Statistical models

#### Variance components and genomic heritabilities

A genomic animal model was applied to estimate variance components for milk production and TSCS. In this regard, we used the AIREML algorithm as implemented in AIREMLf90 from the BLUPf90 package [19]. The statistical model in matrix notation was:

y=Xb+Zg+e[modelVC_nHS]

where **y** = a vector of observations for the test-day or the lactation records; **b** = a vector of fixed effects including herd, calving year, calving month, age at first calving classes, lactation-calving-age classes for dams, classes for days in milk (for the test-day traits), lactation length classes (for lactation traits) and a dummy variable indicating the presence of HS during 1 to 8 weeks before birth for the genotyped cows; **g** = a vector of random additive genetic effects following *N*(0, $G\sigma_{g}^{2}$), where **G** = the genomic relationship matrix constructed according to VanRaden [20] and $\sigma_{g}^{2}$ = the genomic variance; and **e** = a vector of random residuals following *N*(0, $I\sigma_{e}^{2}$), where **I** = an identity matrix and $\sigma_{e}^{2}$ = residual variance. **X** and **Z** were incidence matrices for **b** and **g**, respectively.

Age at first calving was grouped into 6 classes (class 1: ≤ 22 months, class 2: 23–24 months, class 3: 25–26 months, class 4: 27–28 months, class 5: 29–30 months, and class 6: ≥ 31 months). Calving age and lactation (**L**) of the dams were combined to form 21 classes (class 1–6: the same as criteria for age at first calving in L1, class 7: ≤ 35 months in L2, class 8: 36–37 months in L2, class 9: 38–39 months in L2, class 10: 40–42 months in L2, class 11: ≥ 43 months in L2, class 12: ≤ 48 months in L3, class 13: 49–51 months in L3, class 14: 52–54 months in L3, class 15: ≥ 55 months in L3, class 16: ≤ 61 months in L4, class 17: 62–66 months in L4, class 18: ≥ 67 months in L4, class 19: ≤ 77 months in L5, class 20: ≥ 78 months in L5, and class 21: dams in L6). For DIM on the first test-day, 6 classes were defined: class 1: ≤ 10 days, class 2: 11–15 days, class 3: 16–20 days, class 4: 21–25 days, class 5: 26–30 days, and class 6: ≥ 31 days. Lactation length for lactation production traits was classified into 16 classes considering equal intervals between 275 to 305 days. The dummy variable indicating in-utero HS was 1 for the respective week for THI ≥ 60, otherwise, a 0 was assigned. We performed separate runs for each week before calving.

Alternatively, an interaction model (as introduced by Yao et al. [23]) considering interactions between genotype and in-utero HS, was defined:

$$y=Xb+Zg+Wg_{hs}+e\;[model\;VC\_wHS]$$

where **g**_**hs**_ = a vector of genotype by HS interaction effects for cows with in-utero HS following *N*(0, $G_{hs}\sigma_{g_{hs}}^{2}$), where **G**_**hs**_ = the genomic relationship matrix for the cows with in-utero HS and $\sigma_{g_{hs}}^{2}$ = the variance of GxE; **W** = a design matrix allocating phenotypic records to **g**_**hs**_. The remaining effects are the same as described in model VC_wHS.

The genomic heritability ($h_{g}^{2}$) for each trait was calculated as $h_{g}^{2}=\sigma_{g}^{2}/\left(\sigma_{g}^{2}+\sigma_{e}^{2}\right)$ for estimates from model VC_nHS. For estimates from model VC_wHS, the heritability of the common genomic effects ($h_{c}^{2}$) was $h_{c}^{2}=\sigma_{g}^{2}/\left(\sigma_{g}^{2}+\sigma_{g_{hs}}^{2}+\sigma_{e}^{2}\right)$ and the ratio of the variance for genotype by HS interaction effects (*r*_*hs*_) was $r_{hs}=\sigma_{g_{hs}}^{2}/\left(\sigma_{g}^{2}+\sigma_{g_{hs}}^{2}+\sigma_{e}^{2}\right)$.

#### Genome wide associations

Subsequently, we estimated main and interaction effects for every SNP via generalized least squares (GLS) equations according to the algorithm as introduced by Yang et al. [24]. The models to estimate main and interaction SNP effects were:

$$y=Xb+x_{snpi}b_{snpi}+x_{interi}b_{interi}+Zg+e\;[model\;GWA\_nHS]$$

$$y=Xb+x_{snpi}b_{snpi}+x_{interi}b_{interi}+Zg+Wg_{hs}+e\;[model\;GWA\_wHS]$$

For solving the equations of model GWA_nHS, we considered the variance components estimated from model VC_nHS (algorithm is specified below). For solving the equations of model GWA_wHS, we considered the variance components estimated from model VC_wHS (algorithm is specified below).

Matrices and vectors of models for genome wide associations were as follows: **x**_**snpi**_ = a vector of centered genotypes calculated as **m**_**snpi**_−2**p**_**snpi**_ (**m**_**snpi**_ = a vector of genotypes for marker *i* considering all genotyped animals; **p**_**snpi**_ = a vector of allele frequency for marker *i*); *b*_*snpi*_ = a regression coefficient for the *i*^*th*^ SNP (the main SNP effect); **x**_**interi**_ = a vector of centered genotypes for cows undergoing in-utero HS in the respective week before birth (consecutive runs for the 8 different weeks); and *b*_*interi*_ = a regression coefficient for the *i*^*th*^ SNP (the interaction effect) under in-utero HS. The remaining effects, vectors and matrices were identical with the effects as specified for models VC_nHS and VC_wHS.

For GWAS models, E(**y**) = **Xb** + **x**_**snpi**_*b*_*snpi*_ + **x**_**interi**_*b*_*interi*_. Var(**y**) equals to $Var(Zg+e)=ZG\sigma_{g}^{2}Z’+I\sigma_{e}^{2}=G\sigma_{g}^{2}+I\sigma_{e}^{2}$ for model GWA_nHS and to $Var(Zg+Wg_{hs}+e)=ZG\sigma_{g}^{2}Z’+WG_{hs}\sigma_{g_{hs}}^{2}W’+I\sigma_{e}^{2}=G\sigma_{g}^{2}+WG_{hs}\sigma_{g_{hs}}^{2}W’+I\sigma_{e}^{2}$ for model GWA_wHS. **Z** was an identity matrix, because all phenotyped animals were genotyped.

In a self-written R program “GWAInter.R” (in S1 Appendix), we applied GLS to estimate the main and interaction effects for each marker, implying a loop with 41,139 repetitions for all SNP markers. The detailed procedure was:

1. Setting $b_{1}=[b\;b_{snpi}\;b_{interi}]$ and $X_{1}=[X\;x_{snpi}\;x_{interi}],$ then $\hat{b_{1}}={\left(X_{1}^{\prime }V^{-1}X_{1}\right)}^{-1}X_{1}^{\prime }V^{-1}y$ (the last two fixed effects in the solution from GLS were main SNP and interaction effects for marker *i*, respectively) and $\mathrm{var}(\hat{b_{1}})$ = ${\left(X_{1}^{\prime }V^{-1}X_{1}\right)}^{-1}$, **V** = $G\sigma_{g}^{2}+I\sigma_{e}^{2}$ or **V** = $G\sigma_{g}^{2}+WG_{hs}\sigma_{g_{hs}}^{2}W’+I\sigma_{e}^{2}$ (the last two variances were the variances for main SNP and interaction effects for marker *i*, respectively);
2. The test statistic for the *i*^*th*^ main SNP effect was: $\chi_{snpi}^{2}=\frac{{\hat{b_{snpi}}}^{2}}{var(\hat{b_{snpi}})}$, with 1 degree of freedom (df);
3. The test statistic for the *i*^*th*^ interaction effect was: $\chi_{interi}^{2}=\frac{{\hat{b_{interi}}}^{2}}{var(\hat{b_{interi}})}$, with 1 df.

The inflation factor (λ) was calculated based on the chi-squared statistics as: $\hat{\lambda }=\frac{median(\chi_{i}^{2})}{\chi_{0.5,df=1}^{2}}$, where $\chi_{i}^{2}=\chi_{snpi}^{2}$ or $\chi_{interi}^{2}$, and $\chi_{0.5,df=1}^{2}$ = 0.4549 (the statistic for a probability of 0.5 from a chi-squared distribution with 1 df). The *P*-value of the main and interaction effects for each SNP were determined by $\chi_{snpi}^{2}$ and $\chi_{interi}^{2}$, respectively. Significantly associated SNPs were detected according to the Bonferroni correction calculated as *P*_*Bonf*_  =  0.05/(number of SNPs) = 0.05/41,139 = 1.22 × 10^−6^ (-log_10_(*P*_*Bonf*_ )) = 5.92. Additionally, a less stringent correction was applied and defined as suggestive threshold with *P*_*sugg*_ = 0.05/(number of independent SNPs) = 0.05/3873 = 4.76 × 10^−6^ (-log_10_(*P*_*sugg*_)) = 4.89. The number of independent SNPs was calculated based on restrictions for linkage disequilibrium (R^2^ ≤ 0.15) for consecutive genomic windows including 500 SNPs (calculated in PLINK 2.0 [25, 26]).

### Gene annotation

Suggestive SNPs according to *P*_*sugg*_ were annotated to potential candidate genes as listed in the Ensembl genome database [27] for main and interaction SNP effects. In this regard, we used the BiomaRt R package [28, 29]. Only genes located within a window of ±100 kb of suggestive SNP were considered as potential candidate genes. Afterwards, we submitted the identified potential candidate genes to gene ontology (GO) overrepresentation tests [30], using the GO web-tool [31, 32]. The false discovery rate of *P* < 0.05 was considered to identify overrepresented GO terms for biological processes and reactome pathways. In addition, the windows with suggestive SNPs were mapped with the bovine QTL database through the online Data Analysis Tools [33] to elucidate the phenotypic contributions of the genomic segments. According to Hu et al. [33], the phenotypes were concisely classified into milk, meat and carcass, growth performance, reproduction, exterior and health trait categories.

## Results and discussion

### Variance components

**Fig 1:**
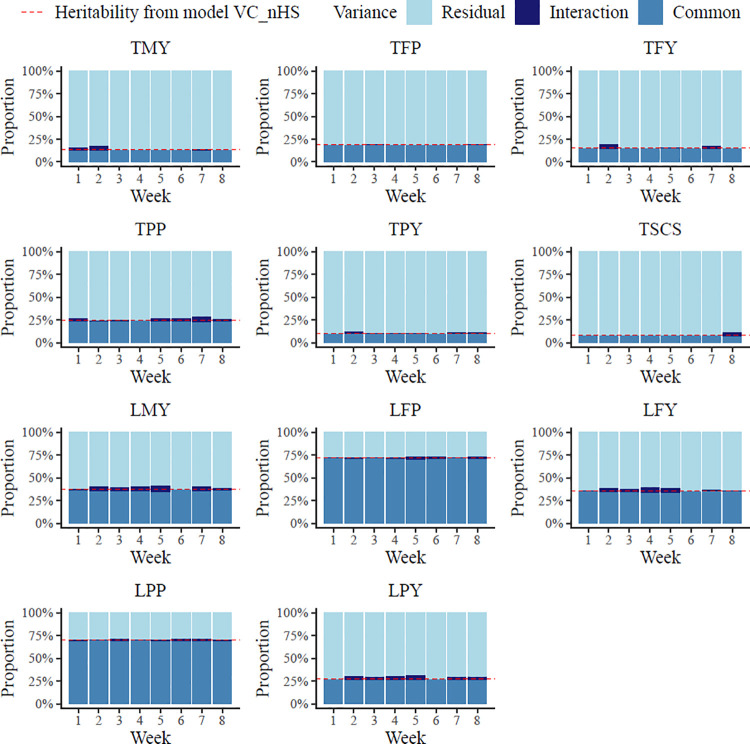
Common genomic, interaction and residual variances of milk production traits and somatic cell score estimated from an animal model with genotype by heat stress interactions during the last eight weeks of pregnancy (model VC_wHS). Dotted line = heritability estimated from an animal model without interaction (model VC_nHS); TMY = first test-day milk yield; TFP = first test-day fat percentage; TFY = first test-day fat yield; TPP = first test-day protein percentage; TPY = first test-day protein yield; TSCS = first test-day somatic cell score; LMY = first lactation milk yield; LFP = first lactation fat percentage; LFY = first lactation fat yield; LPP = first lactation protein percentage; LPY = first lactation protein yield.

The genomic heritabilities from a model without consideration of in-utero HS (model VC_nHS) as well as common genomic, interaction and residual variances estimated from a model with in-utero HS effects (model VC_wHS) are plotted in Fig 1. Basically, common genomic and residual variances were quite constant for all studied weeks during late gestation. Moreover, both variance components from model VC_wHS were approximately the same when compared to the respective estimates from model VC_nHS. Hence, the genomic heritabilities from model VC_nHS and the common genomic heritabilities from model VC_wHS were 0.13 for TMY, 0.19 for TFP, 0.15 for TFY, 0.24 for TPP, 0.10 for TPY, and 0.08 for TSCS. Minor differences in heritability estimates from model VC_nHS and VC_wHS were observed for lactation production traits. In detail, the $h_{c}^{2}$ from model VC_wHS ranged between 0.35 and 0.37 for LMY, between 0.70 and 0.72 for LFP, between 0.34 and 0.36 for LFY, between 0.69 and 0.70 for LPP, and between 0.26 and 0.28 for LPY. The $h_{g}^{2}$ from model VC_nHS for the same traits were 0.37, 0.72, 0.36, 0.71, and 0.28, respectively. However, interaction variances varied to a large extent when HS during last eight weeks of pregnancy was considered in consecutive runs, leading to diverse in-utero HS ratios across the eight weeks. Among all traits, LMY displayed the largest *r*_*hs*_ in a range from 0.00 (WK6) to 0.07 (WK5). The *r*_*hs*_ estimates for TFP were consistently very close to zero in response to climatic impact from all eight last weeks of gestation. For all traits, the interaction variances explained small proportions of the total phenotypic variance. In contrast to small interaction variances reported in our study, environment-specific genomic variances for feed efficiency traits [23] were larger than the common genomic variances from the present studies. However, in this study [23], production and management environments different substantially, because datasets from three countries located in two different continents were merged. Herd specific effects explained very large proportions up to 30% of phenotypic variations in cow production traits in North America [34]. The maximum *r*_*hs*_ of 0.07 in our study indicate that the effect of in-utero HS during late pregnancy is smaller than the herd effect due to geographical impact across latitudes. The time period from in-utero HS measurements until the trait recording date is longer for lactation records, but the *r*_*hs*_ for lactation production traits were slightly larger than for the respective test-day traits. Nevertheless, the stronger impacts might be due to the accumulative in-utero HS effects during the whole lactation.

In general, regarding in-utero HS, WK2 was most important for test-day yield traits, i.e., TMY (*r*_*hs*_ = 0.05), TFY (*r*_*hs*_ = 0.05) and TPY (*r*_*hs*_ = 0.03). For lactation yield traits, WK4 and WK5 displayed the largest *r*_*hs*_, with 0.06 and 0.07 for LMY, 0.06 and 0.05 for LFY, and 0.05 for LPY.

### Inflation factors

**Fig 2:**
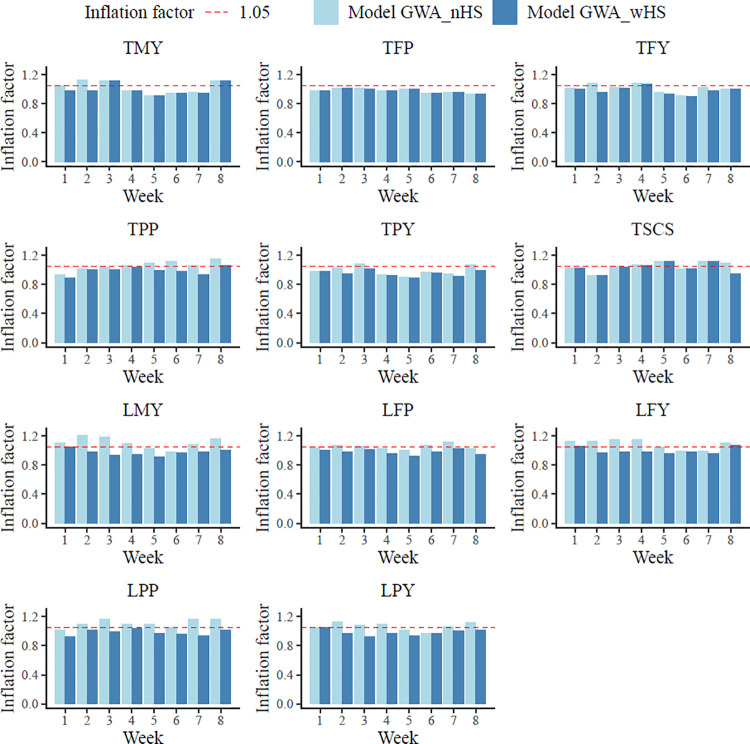
Inflation factors for interaction effects from GWAS models with (GWA_wHS) and without genotype by heat stress interaction (GWA_nHS) during the last eight weeks of pregnancy. Dotted line = inflation factor of 1.05; TMY = first test-day milk yield; TFP = first test-day fat percentage; TFY = first test-day fat yield; TPP = first test-day protein percentage; TPY = first test-day protein yield; TSCS = first test-day somatic cell score; LMY = first lactation milk yield; LFP = first lactation fat percentage; LFY = first lactation fat yield; LPP = first lactation protein percentage; LPY = first lactation protein yield.

The inflation factors for the main effects from model VC_nHS and VC_wHS ranged from 0.74 for LFP to 1.05 for TSCS (S1 Fig). An inflation factor of value 1 represents sufficient correction for the population structure in GWAS, and a value larger than 1.05 indicates overestimation with inflated false positive results [35]. Consideration of the interaction variance in model GWA_wHS had no impact on inflation factors for the main SNP effects (when compared to inflation factors from model GWA_nHS). However, a larger number of values exceeding 1.05 were observed for the SNP interaction effects from model GWA_nHS (Fig 2). Generally, with regard to model GWA_nHS, the inflation factors indicated more false positives for lactation compared to test-day traits. For test-day traits, less than 30% of all runs had a λ > 1.05, but 67.5% of all runs for lactation milk-production traits displayed such an increased inflation factor. Interestingly, when considering the interaction variances for in-utero HS in model GWA_wHS, inflated results decreased to 12.5% for test-day traits and to 5% for lactation traits. Due to the improvements from model GWA_wHS (i.e., the preferable inflation factors), only significant and suggestive SNPs for main and interaction effects from model GWA_wHS were considered in the ongoing gene annotations.

### Significant and suggestive SNPs for main effects

For the main effects, suggestive SNPs from model GWA_nHS overlapped to a large extent with suggestive SNPs from model GWA_wHS. Therefore, only results from GWA_wHS are presented. A total of 380 suggestive SNPs and 237 significant SNPs (including repetitive SNPs for different traits) exceeded the respective thresholds (Table 3). Number of suggestive and significant SNPs equaled to 171 and 98, respectively, when considering only unique SNPs. Detailed information for the suggestive SNPs is given in S1 Table. Among the eleven analyzed traits, LPP displayed the largest number of suggestive SNPs (91), while only three suggestive SNPs were detected for TSCS, indicating the strong polygenic genetic architecture of TSCS compared to milk production traits [36]. For both test-day and lactation records, results for fat and protein percentages indicated a likewise oligo-genic genetic architecture, as previously reported in genomic studies comparing percentage and yield traits [37]. Hence, the number of suggestive SNPs for milk composition traits including TFP, TPP, LFP, and LPP were always larger than for the respective yield traits TFY, TPY, LFY and LPY. It is well known that fat and protein percentages are controlled by some major genes with large effects, and many unknown genes with small effects. In this regard, the gene *DGAT1* on BTA14 strongly contributed to milk fat content [38], and the bovine *ABCG2* gene on BTA6 had a major effect on milk protein content [39]. Accordingly, BTA14 comprised the most suggestive SNPs for all traits, apart from TPY and TSCS. SNPs on BTA6 were suggestively associated with six traits, including TMY, TFY, TPP, LMY, LPP and LPY. A larger number of SNPs were detected for lactation than for corresponding test-day milk production traits, reflecting the larger variation for accumulated lactation than for single test-day traits.

**Table 3:** Number of SNPs with suggestive main effects associated with milk production traits and somatic cell score.

Trait^a^	#sug. SNPs	Chromosome (#sug. SNPs on chromosome)
**TMY**	9	6 (3); 14 (4); 16 (1); 20 (1)
**TFP**	51	3 (1); 14 (40); 27 (10)
**TFY**	27	6 (2); 14 (23); 20 (1); 27 (1)
**TPP**	30	1 (1); 3 (3); 6 (9); 10 (1); 14 (9); 20 (1); 23 (6)
**TPY**	5	9 (1); 10 (1); 11 (2); 20 (1)
**TSCS**	3	5 (1); 24 (2)
**LMY**	34	5 (4); 6(5); 14 (25)
**LFP**	72	3 (1); 5 (24); 14 (43); 20 (4)
**LFY**	37	11 (1); 14 (33); 15 (1); 18 (1); 26 (1)
**LPP**	91	3 (6); 5 (9); 6 (9); 10 (7); 11 (3); 14 (38); 15 (2); 20 (12); 21 (1); 25 (1); 29 (3)
**LPY**	21	6 (8); 11 (2); 14 (9); 20 (1); 24 (1)

^a^: TMY = first test-day milk yield; TFP = first test-day fat percentage; TFY = first test-day fat yield; TPP = first test-day protein percentage; TPY = first test-day protein yield; TSCS = first test-day somatic cell score; LMY = first lactation milk yield; LFP = first lactation fat percentage; LFY = first lactation fat yield; LPP = first lactation protein percentage; LPY = first lactation protein yield.

In general, for milk production traits, the identified suggestive SNPs for main effects, in particular associated SNPs on BTA3, 5, 6, 10, 14, and 20, were in agreement with the associated SNPs as reported in previous studies [40, 41]. A quite large proportion (66.67%) of the suggestively associated SNPs was mapped by significant SNPs influencing milk production traits [40, 41]. For TSCS, Meredith et al. [40, 42] identified dozens of SNPs, but only one QTL region on BTA24 (spanning from 59.67 Mb to 59.76 Mb) was in close proximity to the SNP detected in our study. Meredith et al. [42] used daughter yield deviations for TSCS as phenotypes for sires in their studies. When considering original phenotypes in a cow population as dependent traits, the number of identified SNPs for TSCS decreased to nine [41].

### Significant and suggestive SNPs for interaction effects

With regard to interaction effects, model GWA_nHS generated more suggestive SNPs (51) than model GWA_wHS. This was especially the case for lactation production traits, because the inflation factors for the interaction effects of lactation traits substantially decreased when switching from GWA_nHS to GWA_wHS (Fig 2). The suggestive SNPs from model GWA_nHS contained all suggestive SNPs from model GWA_wHS. Due to the improved inflation factors from GWA_wHS, only suggestive SNPs from GWA_wHS were considered in ongoing gene annotations. The number of suggestive and significant SNPs (31 and 4) for the interaction effects was smaller than for the main effects (Table 4), reflecting the variance proportions for the interaction and common genomic effects, respectively. In contrast to main effects, the number of suggestive and significant SNPs for the interaction effects (estimations from model GWA_wHS) was larger for test-day than for lactation traits. Three from the 31 suggestively associated SNP (model GWA_wHS) were associated with functional traits in Holstein cattle, including *ARS-BFGL-NGS-88748* (at 61.48 Mb on BTA19) with body size [43], *BTB-01195369* (at 90.95 Mb on BTA3) with endocrine fertility traits [44], and *Hapmap51393-BTA-113111* (at 23.81 Mb on BTA8) with cow conception rate [45]. Another three SNPs, i.e., *ARS-BFGL-NGS-17754* (at 52.12 Mb on BTA18), *ARS-BFGL-NGS-73518* (at 90.98 Mb on BTA3) and *ARS-BFGL-NGS-34063* (at 118.09 Mb on BTA5), had an effect on weight, height at withers, and weaning weight, respectively, in Hanwoo cattle [46]. *Hapmap22875-BTA-155031* (at 112.90 Mb on BTA4) was significantly associated with tick resistance in Brazilian Braford and Hereford cattle [47] and *ARS-BFGL-NGS-106479* (at 79.75 Mb on BTA11) with intramuscular fat percentage in Australian beef cattle [48]. Furthermore, the Holstein haplotype of the gene *APOB*, detected as a causal mutation for cholesterol deficiency, embraces the SNP *ARS-BFGL-NGS-106479* [49]. Interestingly, for the interaction effects, the eight SNPs as reported in the aforementioned studies are more relevant for functional than for primary traits in cattle, although our analyses mainly focused on milk production. Results for the suggestive SNP interactions and their associated traits indicate that robust cattle with superior functionality, e.g., favorable fertility and conformation, might be more tolerant to HS during late pregnancy than cattle with breeding focus on milk production. Mapping all suggestive SNPs for interaction effects with the bovine QTL database revealed that the two most important QTL regions were associated with TPP and TPY.

**Table 4:** SNPs with suggestive interaction effects on first test-day milk yield (TMY), fat percentage (TFP), fat yield (TFY), protein percentage (TPP), protein yield (TPY) and somatic cell score (TSCS), and on lactation milk yield (LMY), lactation fat percentage (LFP), lactation fat yield (LFY), lactation protein percentage (LPP) and lactation protein yield (LPY).

Trait	#sug. SNPs	SNP	Week	Chr.	Base pair	MAF	Beta.inter	SE	*P*-value
**TMY**	2	**ARS-BFGL-BAC-33086**	8	19	14537900	0.27	-0.91	0.19	1.47E-06
		ARS-BFGL-NGS-88748	7	19	61476059	0.18	-1.00	0.22	7.99E-06
**TFP**	7	ARS-BFGL-NGS-3794	5	1	89914813	0.13	0.15	0.03	1.37E-06
		Hapmap39006-BTA-116188	1	2	30384603	0.35	-0.10	0.02	6.49E-06
		Hapmap47201-BTA-121648	2	4	91525544	0.39	-0.09	0.02	1.11E-05
		Hapmap22875-BTA-155031	2	4	112899067	0.25	0.11	0.02	3.49E-06
		Hapmap44288-BTA-81147	5,7	8	48282567	0.19	-0.11	0.03	2.61E-07
		ARS-BFGL-NGS-17797	5	18	51967260	0.15	0.14	0.03	1.33E-06
		ARS-BFGL-NGS-17754	5	18	52115086	0.15	0.14	0.03	1.33E-06
**TFY**	3	Hapmap51787-BTA-101971	8	2	54937229	0.23	0.04	0.01	6.09E-06
		Hapmap26991-BTA-151076	8	2	82959890	0.44	0.04	0.01	1.33E-06
		BTA-104962-no-rs	4	6	8553220	0.16	-0.05	0.01	4.31E-06
**TPP**	6	ARS-BFGL-NGS-3821	3	1	60736616	0.32	-0.04	0.01	7.37E-07
		BTB-01195369	8	3	90945010	0.39	-0.04	0.01	7.09E-06
		ARS-BFGL-NGS-73518	8	3	90978652	0.37	0.04	0.01	7.70E-06
		ARS-BFGL-NGS-34063	8	5	118085231	0.33	0.04	0.01	4.97E-06
		ARS-BFGL-NGS-62753	3	12	66123957	0.11	0.06	0.01	7.69E-06
		Hapmap33939-BES5_Contig460_1314	1	24	44888957	0.29	0.04	0.01	9.45E-06
**TPY**	3	ARS-BFGL-NGS-65689	2	8	23528913	0.08	-0.05	0.01	2.00E-06
		Hapmap51393-BTA-113111	2	8	23814719	0.07	0.05	0.01	4.73E-06
		**ARS-BFGL-BAC-33086**	8	19	14537900	0.27	-0.03	0.01	6.19E-06
**TSCS**	4	Hapmap38434-BTA-74956	6	5	106953248	0.19	-0.29	0.06	5.02E-06
		ARS-BFGL-NGS-4427	6	11	81840635	0.14	0.32	0.07	5.48E-06
		ARS-BFGL-NGS-17721	6	11	82176836	0.16	0.30	0.07	1.02E-05
		ARS-BFGL-NGS-20982	1	27	23032376	0.30	0.30	0.05	1.93E-08
**LMY**	3	**ARS-BFGL-NGS-103742**	7,8	10	40203321	0.07	308.38	69.72	9.33E-07
		ARS-BFGL-NGS-106479	4	11	79750751	0.19	-230.38	48.15	1.72E-06
		**ARS-BFGL-NGS-54155**	8	27	7920380	0.37	168.80	38.47	1.15E-05
**LFP**	1	BTB-01985049	3	9	21565055	0.11	-0.07	0.02	7.47E-06
**LFY**	1	ARS-BFGL-NGS-53850	8	21	28216224	0.06	11.58	2.62	1.02E-05
**LPP**	1	BTB-01130157	4	7	56381886	0.25	-0.02	0.00	1.28E-05
**LPY**	3	**ARS-BFGL-NGS-103742**	8	10	40203321	0.07	10.16	2.09	1.24E-06
		**ARS-BFGL-NGS-54155**	8	27	7920380	0.37	5.11	1.16	1.07E-05
		ARS-BFGL-NGS-34282	5	29	33704537	0.41	5.04	1.13	8.03E-06

Week = week before birth; SNP = SNP name; Chr. = chromosome; MAF = minor allele frequency; Beta.inter = interaction effect; SE = standard error.

Bold = SNP with suggestive interaction effects for two traits.

### Potential candidate genes

For test-day milk production traits, a total of 373 unique potential candidate genes are located in regions spanning 100 kb upstream to downstream of suggestive main SNPs from model GWA_wHS (S1 Table). In addition to the *DGAT1* gene, the annotated genes *CPSF1*, *FOXH1*, *ARHGAP39*, *PPP1R16A*, *GRINA* and *MROH1* were frequently associated with milk production traits [41, 50, 51]. Because of the quite large genetic correlations (≥ 0.75; in S2 Table) between test-day and corresponding lactation traits, the percentage of overlapping candidate genes was larger than 78%. Interestingly, the genes *GHR* and *ABCG2* were only detected as candidate genes for lactation, but not for test-day milk production traits. Only three candidate genes were located in close distance to suggestive SNPs for TSCS. However, no study reported a relationship between the three genes and TSCS. The gene *ABCC9* harbors the most significant SNPs for production traits [41], the gene *WDR7* is associated with sperm motility and concentration in Holstein-Friesian bulls [52], and the gene *TXNL1* has an effect on moisture content in rainbow trout [53]. The response to oxygen-containing compound and the G-proteifan-coupled receptors signaling pathway were significantly detected based on all candidate genes for main effects. The first pathway represents biological processes inducing changes in cell or an organism activities (in terms of movement, secretion, enzyme production or gene expression), due to an oxygen-containing compound stimulus [54]. The second pathway recognizes a broad array of extracellular mediators including cationic amines, lipids, peptides, proteins and sensory agents [55].

Due to the limited number of suggestive SNPs for interaction effects from model GWA_wHS, a smaller number including 62 unique candidate genes in the vicinity of the SNP locations, were identified (± 100 Kb, S3 Table). In human, the members of *ABCA6*-like transporters, *ABCA6*, *ABCA8*, *ABCA9* and *ABCA10*, are all integrated in cholesterol-related pathways, either directly or based on their dynamic regulation upon cholesterol application [56]. Chemokines, including *CCL14* and *CCL16*, play important roles in regulating bovine endometrial functions during early pregnancy [57]. The genes *CCL5*, *HEATR9*, *MMP28*, *GIMAP* family and *VEGFC* activate immune responses [58–62]. Proteins encoded by the genes *GAS2L2* and *NRIP2* participate in growth process [63, 64]. Mutations in the *SCN1A* and *TTC21B* genes cause several diseases [65, 66]. Sanglard et al. [67] reported a strong correlation between *ETNPPL* variants and the top 20 differentially expressed genes in beef calves in response to energy restriction during late gestation. *GPC4* from the same family of the identified *GPC5* and the directly identified gene *RASL10B* were favorably associated with *ETNPPL* [67]. The genes *LSAMP* and *GRM8* are involved in emotional and motivational functions [68, 69]. The *MDGA2* gene encodes novel proteins which regulate neuronal migration and neural development [70]. The *GPC5* gene was identified by Naderi et al. [71], who focused on selection signatures in subpopulations of local dual-purpose black and white cattle from Germany. As a result from another selection signature analyses, the gene *FOCAD* was identified by Alshawi et al. [72] in Iraqi cattle. The gene *C8H9orf85* was detected as a candidate gene for milk production traits in water buffalo [73]. The genes *TULP3*, *IQSEC3* and *FAM49A* were identified as potential candidate genes for milk production traits in US Holstein cattle [41] and for milking speed in French Holstein cattle [74]. The genes *RHNO1* and *FOXM1* are linked to a variety of cancers and are defined as DNA damage repair regulators [75]. Demetriou et al. [76] explained the role of the *FKBP4* gene in recurrent fetal losses in humans. Bourneuf et al. [77] identified *de novo* deleterious mutations in the *FAM189A1* gene. The zinc-finger proteins constitute one of the most abundant groups of proteins in the mammalian genome and are involved in several cellular processes, differentiations of serval tissues, development of several diseases, as well as in tumorigenesis, cancer progression and metastasis formation [78].

From the overrepresentation test, two biological processes, including lymphocyte chemotaxis and monocyte chemotaxis, were excessively represented, referring to all genes from the bovine genome. Lymphocytes contribute to the development of immunity and to allergic inflammation of the lower respiratory tract [79]. Monocytes participate in both innate and adaptive immune responses, because of their phagocytic activity [80]. The identified annotations indicate the biological complexity of in-utero HS on productivity in offspring. Nevertheless, sensitivity to in-utero HS in offspring seems to be correlated with the health status of their dams.

In conclusion, the *r*_*hs*_ in the range from 0 to 0.07 indicate quite small effects of HS during late pregnancy on test-day and lactation milk production traits. The accumulative effects due to in-utero HS may explain the generally larger interaction variances for lactation compared to test-day traits. Superiority in inflation factors for the SNP interaction effects from the model GWA_wHS over the model GWA_nHS suggests modelling of interaction variances when applying a GWAS with GxE interactions. Suggestive SNPs for the main effects and their corresponding annotated potential candidate genes reflect findings from previous GWAS focussing on milk production traits. For the SNP interaction effects, eight out of 31 suggestive SNPs are known to effect cattle functionality, indicating a possible favourable correlation between functional traits and heat tolerance. Furthermore, two biological processes as inferred on the basis of the identified 62 candidate genes located in close proximity to the SNPs with suggestive interaction effects, contribute to immune response mechanisms.

## Supporting information

S1 Table Suggestive SNPs and potential candidate genes associated with main effects for test-day and lactation production traits. (XLSX)

S2 Table Genetic correlations between test-day and lactation production traits. (DOCX)

S3 Table Potential candidate genes associated with interaction effects for test-day and lactation production traits. ^a^: TMY = first test-day milk yield; TFP = first test-day fat percentage; TFY = first test-day fat yield; TPP = first test-day protein percentage; TPY = first test-day protein yield; TSCS = first test-day somatic cell score; LMY = first lactation milk.
(XLSX)

S1 Appendix An R script to perform genome wide associations considering interaction between each marker and a discrete environmental descriptor with two classes. (R)

S1 Fig Inflation factors for main effects from GWAS models with (GWA_wHS) and without genotype by heat stress interaction (GWA_nHS) during the last eight weeks of pregnancy. Dotted line = inflation factor of 1.05; TMY = first test-day milk yield; TFP = first test-day fat percentage; TFY = first test-day fat yield; TPP = first test-day protein percentage; TPY = first test-day protein yield; TSCS = first test-day somatic cell score; LMY = first lactation milk yield; LFP = first lactation fat percentage; LFY = first lactation fat yield; LPP = first lactation protein percentage; LPY = first lactation protein yield.
(TIF)
