# Supplementary material for: Identification of candidate genes on the basis of SNP by time-lagged heat stress interactions for milk production traits in German Holstein cattle
Source: PLoS One. 2021 Oct 14;16(10):e0258216. doi: 10.1371/journal.pone.0258216 (PMC8516222; doi:10.1371/journal.pone.0258216)
Supplement: S2 Table — (DOCX) [file pone.0258216.s002.docx]

| **Test-day trait^a^** | **Lactation trait^b^** | **Genetic correlation** | **Standard error** |
| --- | --- | --- | --- |
| **TMY** | **LMY** | 0.82 | 0.02 |
| **TFP** | **LFP** | 0.76 | 0.02 |
| **TFY** | **LFY** | 0.80 | 0.02 |
| **TPP** | **LPP** | 0.75 | 0.02 |
| **TPY** | **LPY** | 0.85 | 0.03 |

^a^: TMY = first test-day milk yield; TFP = first test-day fat percentage; TFY = first test-day fat yield; TPP = first test-day protein percentage; TPY = first test-day protein yield.

^b^:LMY = first lactation milk yield; LFP = first lactation fat percentage; LFY = first lactation fat yield; LPP = first lactation protein percentage; LPY = first lactation protein yield.
