# Supplementary figures and images for: Identification of candidate genes on the basis of SNP by time-lagged heat stress interactions for milk production traits in German Holstein cattle
Source: PLoS One. 2021 Oct 14;16(10):e0258216. doi: 10.1371/journal.pone.0258216 (PMC8516222; doi:10.1371/journal.pone.0258216)

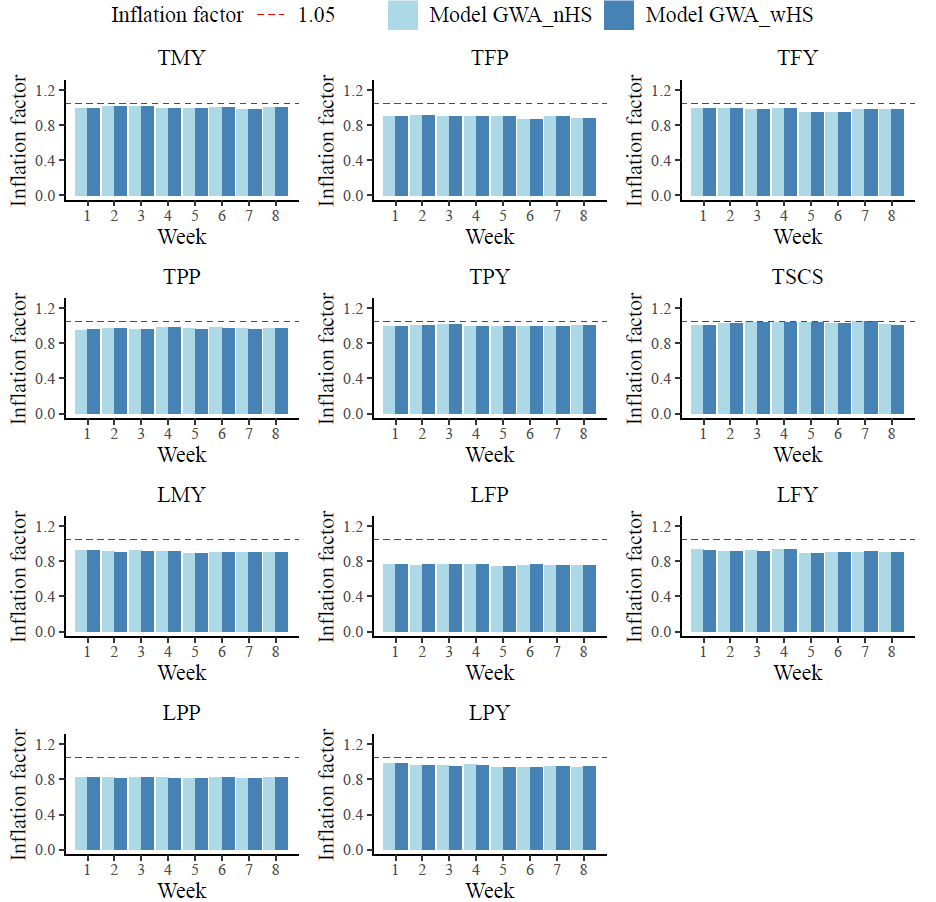

Supplement: S1 Fig — Dotted line = inflation factor of 1.05; TMY = first test-day milk yield; TFP = first test-day fat percentage; TFY = first test-day fat yield; TPP = first test-day protein percentage; TPY = first test-day protein yield; TSCS = first test-day somatic cell score; LMY = first lactation milk yield; LFP = first lactation fat percentage; LFY = first lactation fat yield; LPP = first lactation protein percentage; LPY = first lactation protein yield. (TIF) [file pone.0258216.s005.tif]
